# Role of Sticky Bone in the Management of Various Alveolar Bone Defects: A Systematic Review

**DOI:** 10.7759/cureus.63561

**Published:** 2024-07-01

**Authors:** Vidhuta Sareen, Santhi K, Isha Saxena, Uvashri Selvaraj, Vijayabharathi P, Shalini Chauhan, Gunasekaran M

**Affiliations:** 1 Dentistry, Sri Guru Ram Das Institute of Dental Sciences and Research, Amritsar, IND; 2 Pediatrics, Karur Government Medical College and Hospital, Karur, IND; 3 Prosthodontics, Inderprastha Dental College and Hospital, Ghaziabad, IND; 4 Dentistry, Postgraduate Institute of Medical Education and Research, Chandigarh, IND; 5 Dentistry, All India Institute of Medical Sciences, Raipur, IND; 6 Dentistry, Inderprastha Dental College and Hospital, Ghaziabad, IND; 7 Orthodontics and Dentofacial Orthopedics, Vinayaka Mission’s Sankarachariyar Dental College, Vinayaka Mission’s Research Foundation (Deemed to be University), Salem, IND

**Keywords:** systematic review, bone graft, ridge augmentation, regenerative material, oral surgery, growth factors, alveolar bone defects, sticky bone

## Abstract

Aim: This systematic review aimed to evaluate the effectiveness of sticky bone in managing various alveolar bone defects, examining both its benefits and drawbacks.

Materials and methods: The review adhered to PRISMA guidelines and employed a thorough search strategy using major databases, medical subject headings (MeSH) keywords, and Boolean operators. As a result, the systematic review identified 12 studies focusing on the efficacy of sticky bone in treating alveolar bone defects. Inclusion criteria consisted of randomized controlled trials and case series reporting on the outcomes of sticky bone use for bone defect treatment. Two examiners meticulously performed screening, data extraction, and bias assessment, with the risk of bias evaluated using the Cochrane tool.

Result: The findings indicated significant improvements in bone quality, width, height, and volume, with enhanced predictability in socket preservation and implant placement. Sticky bone was particularly effective in ridge augmentation, guided bone regeneration, and filling periodontal defects, often outperforming alternatives like concentrated growth factors (CGFs) and autologous fibrin glue (AFG). It simplified procedures and reduced resorption during healing, underscoring its value as a versatile adjunct in bone reconstruction surgery.

Conclusion: Sticky bone demonstrated exceptional results in various oral surgeries, effectively addressing issues such as furcation defects, bone loss, and ridge augmentation, with significant clinical and radiographic improvements. Further research is needed to explore its full potential and refine protocols for broader oral surgery and periodontics applications.

## Introduction and background

Oral surgeries aim to enhance healing while minimizing invasiveness, and restoring tissues to their proper structure and function [1]. Rich in growth factors, platelets are crucial for wound healing [2]. Platelet aggregates have shown promise in expediting tissue regeneration in medical and dental contexts [3].

Platelet concentrates like platelet-rich plasma (PRP) and platelet-rich fibrin (PRF) yield various growth factor derivatives [4,5], merging fibrin sealant qualities with platelet-derived growth factors (PDGFs) for optimal tissue regeneration and wound healing [5]. Sticky bone, which combines autologous fibrin glue (AFG) with a bone graft, has become a standard in regenerative techniques [6]. It stabilizes bone grafts, accelerates tissue healing, and reduces bone loss [7].

"Sticky bone" is a composite biomaterial designed for bone regeneration, combining particulate bone substitutes with autologous platelet aggregates, such as PRF and concentrated growth factors (CGFs) [8]. This adaptable material conforms to various bony defects, preventing graft movement and preserving bone volume during healing, thus minimizing the need for block bone and titanium mesh. Its fibrin network captures platelets and leukocytes, releasing growth factors that accelerate bone and soft tissue regeneration without requiring biochemical additives [9]. Additionally, the fibrin interconnection prevents soft tissue ingrowth, making sticky bone suitable for treating intra-bony defects, furcation defects, ridge augmentation, and edentulous alveolar ridge defects [9]. Though studies are limited, a systematic review supports its effectiveness in improving the density and quality of regenerated bone tissue.

## Review

Materials and methods

Our present review was prepared according to the PRISMA guidelines [10]. In our systematic review, the research methodology was made based on the recommendations of the Cochrane Handbook for Systematic Reviews of Interventions [11]. This systematic review is registered in the International Prospective Register of Systematic Reviews (PROSPERO) with the ID: CRD42024534711.

Study question

Is sticky bone effective in the management of various alveolar bone defects, and if so, what are the favorable and unfavorable outcomes of using sticky bone to correct bone defects?

Search strategy

In this review, we conducted a comprehensive cross-disciplinary search, including randomized controlled trials and case series, to evaluate the efficacy of sticky bone in managing alveolar bone defects. We sourced information from original articles, systematic reviews, and relevant citations and bibliographies on the topic of sticky bone's role in treating such defects.

**Figure 1:**
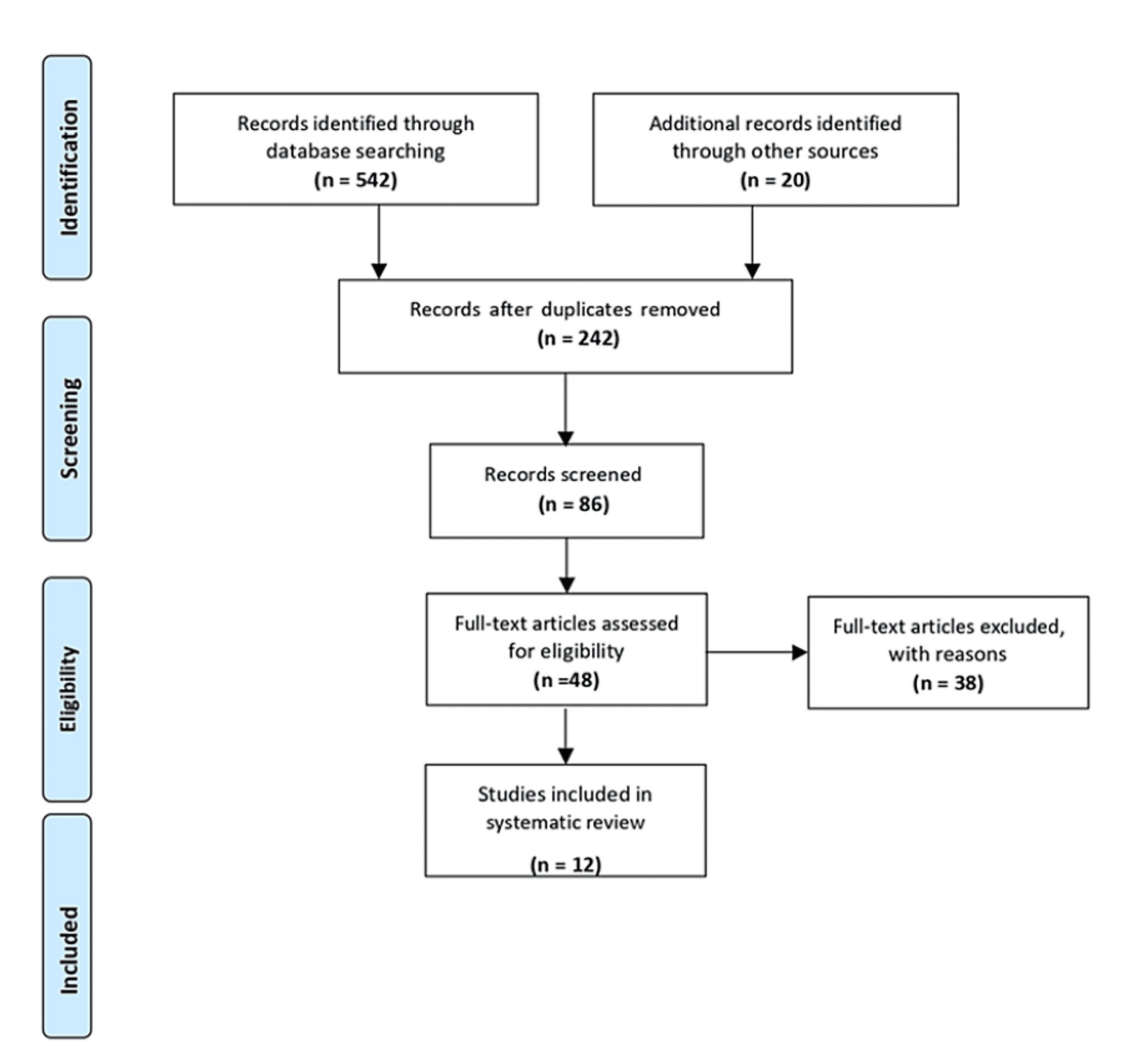
PRISMA flowchart From reference [10]

Our search, conducted up to December 2023, was extensive and encompassed major databases like PubMed, Embase, Web of Science, and Google Scholar. We used medical subject headings (MeSH) keywords and Boolean operators to refine our search strategy (Table 1) and adhered to the PRISMA statement guidelines (Figure 1).

**Table 1:** Search strategy using various keywords in multiple search databases MeSH: medical subject headings

Search database	Keywords and Boolean operators
PubMed	MeSH words “sticky bone”, “autologous fibrin glue”, “alveolar bone defect”, “alveolar ridge defect”, “ridge augmentation”, “intra bony defect” with Boolean operators “AND”, “OR”
Embase	Emtree keywords (“sticky bone”/exp OR “autologous fibrin glue”) AND (“alveolar bone defect”/exp OR “alveolar ridge defect”/exp OR “bony defect”/exp OR “intrabony defect”/exp OR “ridge augmentation”)
Web of Science	TS = (sticky bone OR bone graft with autologous fibrin glue) AND TS = (alveolar ridge defect OR alveolar bone defect OR bony defect OR intra bony defect OR ridge augmentation)
Google Scholar	(“sticky bone” OR “bone graft with autologous fibrin glue”) AND (“alveolar bone defect” OR “alveolar ridge defect” OR “bony defect” OR “intra bony defect” OR “ridge augmentation”)

Selection criteria

For our systematic review, we included randomized controlled trials and case series that assessed the effectiveness of sticky bone in treating alveolar bone defects, provided they reported predictive outcomes and specific datasets. We encompassed studies addressing various bone defects treated with sticky bone, including those where it was combined with other materials; however, studies solely focusing on the in vitro use of sticky bone were excluded.

To maintain consistency, we established inclusion and exclusion criteria based on the study format, participant, intervention, comparison, and outcome (Table 2). We excluded unpublished papers, articles lacking full text or presenting only abstracts, as well as those not published in English.

**Table 2:** Study format, participant, intervention, comparison, and outcome (PICO) criteria for this systematic review CBCT: Cone-beam computed tomography; RCT: Randomized controlled trial

Population	Patients having alveolar bone defect and approached for treatment
Intervention	Sticky bone application
Comparison	CBCT, clinical evaluation
Outcome	Increase in quantity and quality of bone in terms of width and height gain
Study format	RCT, case report, and case series

Screening and selection

Two examiners conducted the entire search and screening process. Initially, irrelevant citations were excluded, and then the headings and abstracts of the remaining articles were analyzed to determine their adherence to our inclusion and exclusion criteria. Articles lacking sufficient information were promptly eliminated. Any uncertainties prompted a full download of the article for a comprehensive evaluation, with input from a second reviewer.

The collected articles underwent further scrutiny by both examiners to ensure they met the eligibility criteria. Articles lacking proper design, essential data, or accurate referencing were excluded. Finally, all retained articles were meticulously reviewed, and relevant data were extracted.

Data extraction

The selected articles underwent data extraction by the first examiner, followed by a thorough review by another examiner to ensure accuracy. Data collection was conducted consistently from the approved articles meeting the inclusion criteria.

The obtained data was standardized and electronically formatted using Office Excel 2013 software (Microsoft® Corp., Redmond, WA, USA). Information was organized with headings such as author, year, study type, objective, materials used, study group, sample size, evaluation method, type of defect, outcome, and conclusion (Table 3).

**Table 3:** Data extraction and categorization of studies included in the systematic review RCT: Randomized controlled trials; CGFs: Concentrated growth factors; PRF: Platelet-rich fibrin; i-PRF: Injectable platelet-rich fibrin; CBCT: Cone-beam computed tomography; GTR: Guided tissue regeneration; ABBM: Anorganic bovine bone matrix; AFG: Autologous fibrin glue; PPD: Probing pocket depth; CAL: Clinical attachment loss; GBR: Guided bone regeneration; OPG: Orthopantomogram

Author	Type of study	Objective	Material used	Study group	Sample size	Evaluation method	Type of defect	Outcome	Inference
Atia et al. [9]	Case series	To evaluate the efficacy of sticky bone and CGF-enriched fibrin membrane in the management of dehiscence defects around dental implants in the narrow maxillary anterior ridge	Autologous CGF-enriched bone graft matrix (sticky bone) and CGF-enriched fibrin membrane	Patients undergoing implants presenting upper alveolar ridge width less than 4 mm	11	CBCT	Vertical labial dehiscence	Vertical dehiscence defect was sufficiently recovered in 5 implant sites while in the other 6 sites, it was decreased to a mean value of 1.25 ± 0.69 mm	This combination increases the quality (density) of the newly formed bone and enhances the rate of new bone formation
Joshi et al. [12]	Case report	To assess the efficacy of ridge augmentation with sticky bone	Sticky bone: a combination of human tooth allograft and autologous fibrin glue	Patient with class III alveolar ridge deficiency in the right maxillary canine region	1 (43-year adult)	CBCT, clinical evaluation	Alveolar ridge deficiency	Substantial gain in alveolar bone width as well as height	Human tooth allograft mixed with autologous platelet, concentrates help in improving alveolar bone profile
Romesh et al. [13]	Case report	To demonstrate the use of sticky bone and autologous PRF membrane in hard tissue regeneration and maintenance of volume of alveolar ridge to enable dental implant placement	Sticky bone formed by cera bone granules of particle size 0.5-1.0 mm and PRF membranes	The patient has a large defect area in the canine region due to a previously impacted canine and requires an implant	1 (22- year adult)	OPG, CBCT	Bony defect with loss of bone due to impaction	Sufficient bone volume was achieved through GBR as shown by comparing presurgical and post-three months CT and clinically	This resulted in a successful GBR, thus augmenting the alveolar ridge defect
Dong-Seok et al. [14]	Case report	To assess the effectiveness of simplified ridge augmentation using sticky bone	Sticky bone prepared by mixing 1.5 cc porcine bone with autologous fibrin glue	Patient with masticatory difficulty on the lower right edentulous ridge and seeking implant-supported restoration	1 (65-year female)	Clinical evaluation	Severe horizontal and vertical alveolar defect in edentulous mandible	Sticky bone prevented collapse of the bone graft and minimized graft resorption during healing	A new innovative surgical procedure by him is more successful and simplified the ridge augmentation
Darwish et al. [15]	RCT	To assess socket preservation of the alveolar ridge by an organic bovine bone and autogenous particulate bone vs sticky bone	An organic bovine bone and autogenous particulate bone vs mixture of injectable platelet-rich fibrin, ABB, and autogenous particulate (sticky bone)	Patients seeking implant rehabilitation who suffered from non-restorable mandibular molars	20	CBCT	Bony defect in lower posterior alveolar ridge region	Sticky bone showed a statistically significant higher mean value of bone area percent compared to other group	Sticky bone showed increased predictability in preserving the socket from collapse which in turn resulted in successful implant placement
Beneytout et al. [16]	Retrospective case series	To assess the effectiveness of guided bone regeneration using platelet-rich fibrin membrane and sticky bone graft	Platelet-rich fibrin membrane and sticky bone graft	Patients with a mean age of 58.2 ± 8.9 years with volumetric bone defects on the maxilla	6	CBCT	Volumetric bony defects	The average height gain was 5.7 ± 2.1 (2 to 8) for the vestibular wall and 3.3 ± 1.2 (2 to 5) for the palatine wall. The average gain in crestal width was 3 to 5 mm	This is more effective in the healing of hard and soft tissues and acts as a supplement in bone reconstruction surgery
Ghoderao et al. [17]	RCT	To evaluate and compare the effects of sticky bone and concentrated growth factors in the treatment of intrabony osseous defects	Sticky bone and CGFs	Patients with 40 intra bony defects	20	CBCT and clinical evaluation at baseline and 6- and 12-months post-therapy	Intra bony defects	Significant reduction of probing pocket depth and gain in clinical attachment level in both the study groups	Intrabony defects treated with sticky bone showed improved clinical and radiographic parameters, indicative of enhanced periodontal regeneration
Soni et al. [18]	Case report	To assess the efficacy of sticky bone and GTR membrane in periodontal intrabony defect	Sticky bone and GTR membrane	Patient with periodontal pocket in relation to lower canine and premolar	1 (58-year adult)	CBCT and clinical evaluation	Intrabony defect	A reduction in the probing pocket depth from 7 mm (pre-operative) to 3 mm, and CAL from 8 mm to 4 mm	Significant improvement in clinical parameters such as PPD, CAL, and radiographic bone fill indicates the success of regenerative therapy using i-PRF with bone grafts
Tony et al. [19]	Randomized parallel arm clinical trial	To evaluate the efficacy of sticky bone in horizontal ridge augmentation with and without collagen membrane	Group 1: Injectable platelet-rich fibrin with sticky bone Group 2: Sticky bone only	Patients with partially edentulous ridges	20 with 10 per group	CBCT before treatment and after 6 months	Deficient edentulous ridges	Horizontal ridge width mean gains of 1.35 mm, 1.55 mm, and 1.93 mm at three levels (crest, 3 mm, and 6 mm) in Group I and 2.7 mm, 2.8 mm, and 2.6 mm at three levels in Group II	Sticky bone (xenogenic bone graft + i-PRF) served as a promising biomaterial in achieving better horizontal bone width gain
Parthasarathy et al. [20]	Case series	To evaluate the clinical efficacy of sticky bone in horizontal ridge augmentation procedures	Sticky bone which is an ABBM combined with i-PRF	Patients with partially edentulous ridges	10	CBCT and clinical evaluation	Partially edentulous bone defect	There was a statistically significant improvement in horizontal ridge width, vertical bone height, and keratinized tissue width	Sticky bone may be used as a promising biomaterial in achieving significant horizontal bone width gain
Abdelhamid et al. [21]	Prospective RCT study	To assess implant stability and radiographic outcomes using AFG against sticky bone combined with crestal sinus lift procedure and simultaneous implant insertion	AFG against sticky bone	Patients with remaining bone height ranging from 5 to 9 mm, with a patent maxillary sinus ostium and missing maxillary posterior tooth	10 patients	Panoramic radiographs, CBCT, and clinical evaluation	Less bone height and patent maxillary sinus ostium	There was no statistical difference between both groups in terms of implant stability, graft apical height, and graft sinus height	AFG and sticky bone are considered safe materials when applied locally
Bhandari et al. [22]	Case report	To treat grade III furcation involvement of mandibular molar with sticky bone and PRF membrane	Sticky bone with PRF membrane	Patient with persistent tooth sensitivity in lower posterior region for 3 months	1 (51-year adult)	CBCT and clinical evaluation	Grade III furcation defect	Quicker tissue healing, significant pocket reduction, clinical attachment gain, as well as radiographic bone fill in both cases	Successful periodontal regeneration of grade III furcation defects can be achieved by using PRF in combination with sticky bone

Assessment of risk of bias

As this review encompassed both randomized controlled trials and case studies, the risk of bias was assessed using the Cochrane risk of bias tool. This tool evaluates seven domains of study methodology, including random sequence generation, allocation concealment, blinding of participants and outcome assessors, completeness of outcome data, selective outcome reporting, and other biases. The risk of bias for each included study was determined (Table 4).

**Table 4:** Assessment of risk of bias

Study	Random sequence generation	Allocation concealment	Blinding of participants	Blinding of outcome assessment	Incomplete outcome data	Selective reporting	Other bias
Atia et al. [9]	+	+	_	+	+	+	+
Joshi et al. [12]	?	_	_	?	+	+	+
Romesh et al. [13]	_	?	_	+	+	?	+
Dong-Seok et al. [14]	?	_	_	+	+	+	+
Darwish et al. [15]	+	+	_	+	+	+	+
Beneytout et al. [16]	+	+	_	?	+	?	+
Ghoderao et al. [17]	+	+	_	+	+	+	+
Soni et al. [18]	_	+	_	?	+	+	_
Tony et al. [19]	+	+	_	+	+	+	+
Parthasarathy et al. [20]	+	+	_	_	+	?	?
Abdelhamid et al. [21]	+	+	_	+	+	+	+
Bhandari et al. [22]	_	+	?	+	+	_	_

Results

Search and Selection

The review's article selection process followed the PRISMA flowchart. A literature search yielded 542 relevant studies, with an additional 20 studies identified through supplementary records. After excluding 242 articles due to irrelevance, duplication, or lack of data, the screening process yielded approximately 86 articles. Of these, 48 full-text articles were assessed against eligibility criteria, resulting in the exclusion of 38 articles with explanations. Ultimately, 12 articles met the criteria and were included in the systematic review (Figure 1).

Description of Studies

This review examines 12 studies on sticky bone's effectiveness in treating various alveolar bone defects. Atia et al. found that sticky bone and CGF-enriched fibrin membrane improved bone quality and formation around dental implants in the narrow maxillary anterior ridge [9]. Joshi et al. showed significant gains in bone width and height with sticky bone ridge augmentation [12]. Romesh et al. reported excellent bone volume improvement using sticky bone and autologous PRF membrane for guided bone regeneration [13]. Dong-Seok et al. suggested a simplified approach to ridge augmentation with sticky bone, reducing resorption during healing [14].

In 2021, Darwish et al. evaluated socket preservation of the alveolar ridge using anorganic bovine bone and autogenous particulate bone versus sticky bone [15]. Their findings suggested increased predictability of socket preservation with sticky bone, facilitating successful implant placement. Beneytout et al. conducted a case series evaluating guided bone regeneration with PRF membrane and sticky bone graft, demonstrating its effectiveness as a supplemental approach in bone reconstruction surgery [16].

In 2022, Ghoderao et al. compared sticky bone and CGFs for intrabony osseous defects, finding superior periodontal regeneration with sticky bone [17]. Similarly, Soni et al. reported significant bone fill-in periodontal intrabony defects using sticky bone and guided tissue regeneration (GTR) membrane [18]. Tony et al. showed promising results for horizontal ridge augmentation with sticky bone, with or without a collagen membrane, leading to better horizontal bone width gain [19]. Parthasarathy et al. further supported sticky bone's efficacy in ridge augmentation through a case series [20].

In 2023, Abdelhamid et al. compared AFG to sticky bone in combination with crestal sinus lift and simultaneous implant insertion, finding favorable outcomes with sticky bone regarding implant stability and radiographic results [21]. Furthermore, Bhandari et al. presented a case report demonstrating successful periodontal regeneration of a grade III furcation defect using sticky bone alongside a PRF membrane [22].

Discussion

In this systematic review, we investigated the efficacy of sticky bone in treating diverse alveolar bone defects, encompassing issues like bone dehiscence, furcation involvement, intrabony defects, ridge augmentation, and loss of alveolar ridge caused by various factors. Our primary aim was to validate the hypothesis that sticky bone significantly contributes to periodontal regeneration and enhances bone quantity and quality in terms of width and height.

Our study stands out as the inaugural systematic review investigating the impact of sticky bone on managing diverse alveolar bony defects, such as horizontal and vertical, as prior reviews on this topic were lacking. We comprehensively analyzed various types of research exploring the application of sticky bone in addressing periodontal defects, regenerative procedures, ridge augmentation, and other alveolar bone issues. Through meticulous screening, we included 12 refined studies that robustly supported our hypothesis concerning the efficacy of sticky bone across a spectrum of alveolar bone defects and periodontal surgeries.

Properties of Sticky Bone

"Sticky bone" is a cutting-edge biomaterial blend that boosts bone regeneration by combining a particulate bone substitute with autologous platelet aggregates, like PRF and CGF [23]. Its adaptable nature is ideal for different bone defects, preserving bone volume during healing and reducing the need for block bone and titanium mesh. The fibrin network in sticky bone captures platelets and leukocytes, speeding up the release of growth factors for faster soft tissue and bone regeneration. Notably, it requires no additional biochemical additives and decreases soft tissue ingrowth into the graft, improving regeneration outcomes [24,25].

Applications of Sticky Bone

As per the systematic review data, the sticky bone showed promising improvement and results in various alveolar bone defects. The CGF-enriched sticky bone, when combined with a CGF-enriched fibrin membrane, can be effective in the reconstruction of dehiscence bone defects formed during implant placement and assist in guided bone regeneration, which is a well-established technique for increasing resorbed alveolar ridges [9]. The improved mean bone density for sticky bone was 1558.9 HU ± 154.2 HU, with a minimum recorded value of 1371.0 HU and a maximum recorded value of 1816.0 HU, as per Atia et al. [9]. It also helped in hard tissue regeneration and maintenance of the volume of the alveolar ridge to enable dental implant placement.

GBR with sticky bone and PRF membrane is more effective for alveolar ridge augmentation, with several advantages such as easy preparation protocols, versatility and biosafety (autologous preparation), cost-effectiveness, enhanced repair and regenerative capacities, and prolonged holding and sustained release of growth factors and proteins [9,13].

Ridge Augmentation With the Help of Sticky Bone

Generally, guided bone regeneration in ridge augmentation procedures often requires a collagen barrier membrane, which is intended to protect and encapsulate the graft material during the sensitive bone-remodeling phase and its integration with the native bone [26]. However, there are several drawbacks to using GBR membranes, such as the difficulty in membrane stabilization, its exorbitant cost, and rapid and unpredictable disintegration, which can result in a weakened barrier effect [27]. A study by Tony et al. proved that sticky bone does not require any collagen membrane necessity, as it alone can assist in horizontal ridge augmentation and facilitate excellent guided bone regeneration [19].

Preservation of Extraction Socket by Using Sticky Bone

The sticky bone can also be used in socket preservation, mainly in older patients after tooth extraction, and provides good bone quality for further implant placement. The placement of sticky bone resulted in grafted material adherence to the recipient sites without micro and macro movements, and the PRF matrix prevented early epithelial ingress onto the defect site, resulting in significant new bone gain in both horizontal and vertical dimensions [28]. Furthermore, the PRF-released growth factors, such as PDGF, epidermal growth factor (EGF), insulin-like growth factor (IGF), fibroblast growth factor (FGF), and vascular endothelial growth factor (VEGF), aid in encouraging cellular proliferation and enhance vascularity in the surgical site [28].

The sticky bone provided better bone quantity and quality in socket preservation after tooth extraction. The sticky bone was compared with anorganic bovine bone in terms of socket preservation of defective alveolar ridge and found that anorganic bovine bone showed a mean area percent of bone of 42.34%, while the sticky bone group showed 57.92% with a significant value (p < 0.05), as per Darwish et al. [15].

Management of Intra-bony Defects With Sticky Bone

The sticky bone can also be packed into furcation, mainly grade III, and intra-bony defects, which showed significant tissue healing, clinical attachment gain, reduction in periodontal probing depth, and radiographic bone fill, as per Bhandari et al. [22]. These changes might have been a result of true periodontal regeneration by means of new attachment, or a long junctional epithelium between the newly regenerated tissues and the root surface. Also, sticky bone showed more promising clinical and radiographic outcomes than CGFs, as proved by Ghoderao et al. [17].

Sticky Bone in Combination With Other Biomaterials for Ridge Augmentation

A study involving 28 patients found that using sticky bone and CGF combined during anterior alveolar horizontal augmentation was more effective than conventional GBR [29]. The three-dimensional architecture and mechanical characteristics of three combinations of autologous platelet liquid (APL), blood, or physiological water, and composite bovine graft, were assessed in a previously conducted in vitro study [30]. The mechanical resistance increased by 875% when APL and bone graft were combined, according to the results. With the creation of a composite sticky graft block and enhanced clinical strategies, this combination of biomaterials could improve the maxillary bone defects treatments. The clinical outcomes of guided bone regeneration using the bone-shell technique and sticky bone in horizontal ridge augmentation were compared in a prior retrospective study. Eighty patients had the bone-shell technique and sticky bone, and CBCT was used to measure the ridge widths before and six months after surgery. Records were maintained on implant survival rate and post-operative complications. The two groups' results were not different significantly, and there was no record of implant failure. Clinical results were comparable for bone-shell technique and sticky bone. Despite the initial complications and patient dropouts, the remaining participants showed consistent improvement in ridge width without significant differences between the test and control groups. Additionally, all implants remained successful over the follow-up period, indicating a positive long-term outcome [31].

Limitations and scope for further research

The major limitation of our systemic review was that it included only four randomized controlled trials, which were present to date and the remaining were case reports and series. Larger studies are needed to evaluate the interest of sticky bone in various oral and periodontal surgical procedures by comparing it with other existing materials and conventional techniques. It is likely that the platelet aggregates all have different and interesting qualities for the healing of soft and hard tissues, Further research and studies should be necessary and validation to be achieved with long‑term follow‑ups.

## Conclusions

Based on current evidence, sticky bone yields outstanding clinical and radiographic results across diverse alveolar bone defects and periodontal surgeries. It has proven effective in addressing furcation defects, infra-bony defects, dehiscence defects, ridge augmentation, and alveolar ridge preservation procedures. Notable outcomes include favorable vertical and horizontal bone gain, clinical attachment improvement, reduced periodontal probing depth, and enhanced radiographic bone fill. Future research is advised to expand on these findings, develop additional protocols, and explore further applications of sticky bone in oral surgical and periodontal procedures.
